# Is it worth trying? A cross-sectional study on the implementation of point-of-care ultrasound in Hungarian primary care

**DOI:** 10.1186/s12875-024-02578-z

**Published:** 2024-09-05

**Authors:** Róbert Kiss-Kovács, Blanka Morvai-Illés, Albert Varga, Gergely Ágoston

**Affiliations:** Department of Family Medicine, Faculty of Medicine, University of Szeged, Szeged, Hungary

**Keywords:** Primary care, General practitioners, Point-of-care ultrasound, Survey, Implementation

## Abstract

**Background:**

Although the number of point-of-care ultrasound devices available in Hungarian primary care practices are increasing due to government funding, their use in day-to-day patient care is limited and unregulated. Our study aimed to evaluate the attitudes and needs of general practitioners (GPs) and patients in Hungary regarding the introduction of bedside ultrasonography in primary care practices.

**Methods:**

As a part of a cross-sectional study, an anonymous, self-administered questionnaire was distributed to GPs and patients on a social media platform. Data collection was carried out from August 2023 to October 2023. Chi-square test was used to determine the associations between categorical variables.

**Results:**

The survey was completed by 415 GPs (mean age 53.8 ± 11.1 years, 54.9% female, mean 19.5 ± 11.9 years of practice) and 693 patients (mean age 45.5 ± 12.3 years, 95.2% female). There was a statistically significant increase in interest in PoCUS among young and middle-aged GPs (age 28–59; *p* = 0.02). In addition, this population of GPs was also more likely to undertake training in PoCUS than their older colleagues (*p* < 0.0001). An inverse relationship was found between the duration of practice and training willingness (*p* = 0.0011). Even with the government’s financial support, only 8.2% of GPs currently use PoCUS in a daily basis, and 59.5% of GPs are unfamiliar with the indications and the ways of using it. Patients would even pay to have the examination done in a primary care setting, even though only 45.9% of patients would pay a GP who is not certified in PoCUS, but the willingness to pay increased to 99.4% for those with formal training (*p* = 0.024).

**Conclusion:**

Our findings indicate a significant interest in adapting PoCUS in primary care from both GPs and patients. Based on the fact that a significant proportion of Hungarian GPs are unaware of PoCUS and its indications, it is particularly important to develop educational frameworks, and practical guidelines for the effective incorporation of PoCUS in Hungary.

**Supplementary Information:**

The online version contains supplementary material available at 10.1186/s12875-024-02578-z.

## Background

Point-of-care ultrasound (PoCUS) is increasingly becoming the ‘stethoscope of modern medicine’ complement to physical examination [1, 2]. Rapid technological advances in recent decades have also led to considerable changes to medical diagnostic tools [3, 4]. Initially, bedside ultrasonography was widely used for the rapid assessment of trauma cases in the 1990s [5]. Since then, in many areas of inpatient care, mobile, hand-held, pocked-sized ultrasound devices, which are ideal for binary decision-making on clinical questions, have been gaining ground for many years. Their use is also important in time-sensitive urgent cases for rapid, accurate, noninvasive diagnosis and prompt initiation of definitive therapy, which facilitates improved clinical outcomes [6–8]. The last decade has seen an expansion of point-of-care ultrasonography in family medicine in several countries, but not at nearly the same pace as in inpatient care [9]. However, several known nations, including Hungary, have not yet spread this imaging technique in the family medicine community. For the United States, the goal is still to introduce uniform PoCUS training for family medicine residents, based on the point-of-care ultrasound training curriculum incorporated and published by the American Academy of Family Physicians in 2016 [10]. This has not yet occurred uniformly due to the lack of implementation of training standardisation, funding challenges and lack of resources [11]. As in the USA, there is considerable diversity in the use of PoCUS among European countries. The reasons for this are manifold and can be attributed to factors that have not yet been resolved [12]. The organisation of primary care varies significantly across Europe, affecting the availability and use of ultrasound. For instance, data from twenty European countries suggest that the accessibility of in-house ultrasound ranges from 0% in England to 98% in Germany, largely due to differences in training, clinic size, and financial incentives. Understanding these substantial differences is crucial for developing effective policies and training programs to improve the integration of ultrasonography in primary care [13]. Several factors can limit the implementation of point-of-care ultrasound in primary care, including insufficient training, time constraints, and limited access to devices, as identified by Andersen et al. and Peng et al. These studies emphasise the need for enhanced training programs and standardised curricula to support PoCUS integration in primary care settings [14, 15]. In Hungary, one such factor that needs to be resolved is the lack of training and qualifications of family physicians and the absence of the implementation of detailed guidelines and a compatible and sustainable training programme in the curriculum for family medicine residents. In addition to training, financing, procurement, legal and quality assurance aspects still need to be clarified [16–18]. These shortcomings must be addressed so that PoCUS can become an integral part of everyday work in GP practices. To overcome these limitations, we considered it necessary to determine the current views of both GPs practising in Hungary and the adult population covered by social security in Hungary on implementing this diagnostic tool. To make it worthwhile to start making changes, it is pivotal to discover the impressions and attitudes of these two populations towards PoCUS.

## Methods

### Study design and participants

As a part of a cross-sectional study, two online, self-administered, anonymous questionnaires were distributed via Facebook. The first nineteen questions, targeted actively on practicing, board certified GPs in a closed medical group with 3.909 members, and was approved by the group administrator. The second survey, consisting of eighteen questions, was directed at patients receiving public healthcare in a closed group specialising in giving medical advice for non-health professional people with 69.011 members, with permission from the group administrator. Both questionnaires featured a mix of open, closed, and semiclosed questions, ensuring a comprehensive and representative outcome. Despite being voluntary, participants’ responses were kept confidential through a cloud-based two-factor authentication security system, safeguarding against unauthorised access. Data collection was carried out from August 2023 to October 2023. During these period, the EUR/HUF exchange rate was 1 EUR to 379.88 HUF.

### Inclusion and exclusion criteria

GPs could complete the questionnaire if they are board certified, and actively practising in a mixed or adult general practice in Hungary during the data collection. Patient eligibility required permanent residence in Hungary, with the entitlement to free, public healthcare benefits. Patients under 18 years of age were excluded. GPs practising outside Hungary and patients without a Hungarian address were also excluded.

### Questionnaires

The questionnaires were developed based on a comprehensive literature review and expert consultations to ensure relevance and credibility. A pilot study with a small group of GPs (n = 10) and patients (n = 10) was conducted to enhance the validity and reliability of the survey. The items differed between patients and GPs to address their distinct perspectives on the subject. While the GPs’ questionnaire focuses mainly on their experiences with PoCUS and their expectations of its education, the questionnaire for patients addresses trust, acceptance, and willingness to pay for PoCUS services. A digital informed consent was obtained from both patients and general practitioners, who participated in the study. Participants were required to consent by clicking on the acknowledgement button before proceeding to the questionnaire (can be found as Supplementary Material). The GPs’ survey included demographic questions (age, sex) and fifteen questions about their practice, as well as their knowledge, training, and views on the use and regulation of PoCUS. Patients also answered demographic questions (age, sex, education, residence) and twelve questions on PoCUS in primary care, including questions about trust, training, availability, satisfaction, willingness to pay, and the importance of patient education and information. Both surveys included open questions on the advantages and disadvantages of using PoCUS.

### Data analysis

Data analysis was performed with IBM^®^ SPSS^®^ software, version 29.0.1.0 (SPSS, Chicago, IL). Descriptive statistical analysis was presented as mean with standard deviation (SD) for continuous variables. Categorical variables were summarised using frequency and percentage. The chi-square test was used to determine the associations between categorical variables. Age and the duration of practice were treated as a categorical variable in the analysis. A p*-*value less than 0.05 was considered significant.

## Results

A total of 415 GPs, and 693 patients completed the questionnaires.

### Results of the questions answered by GPs

**Fig. 1:**
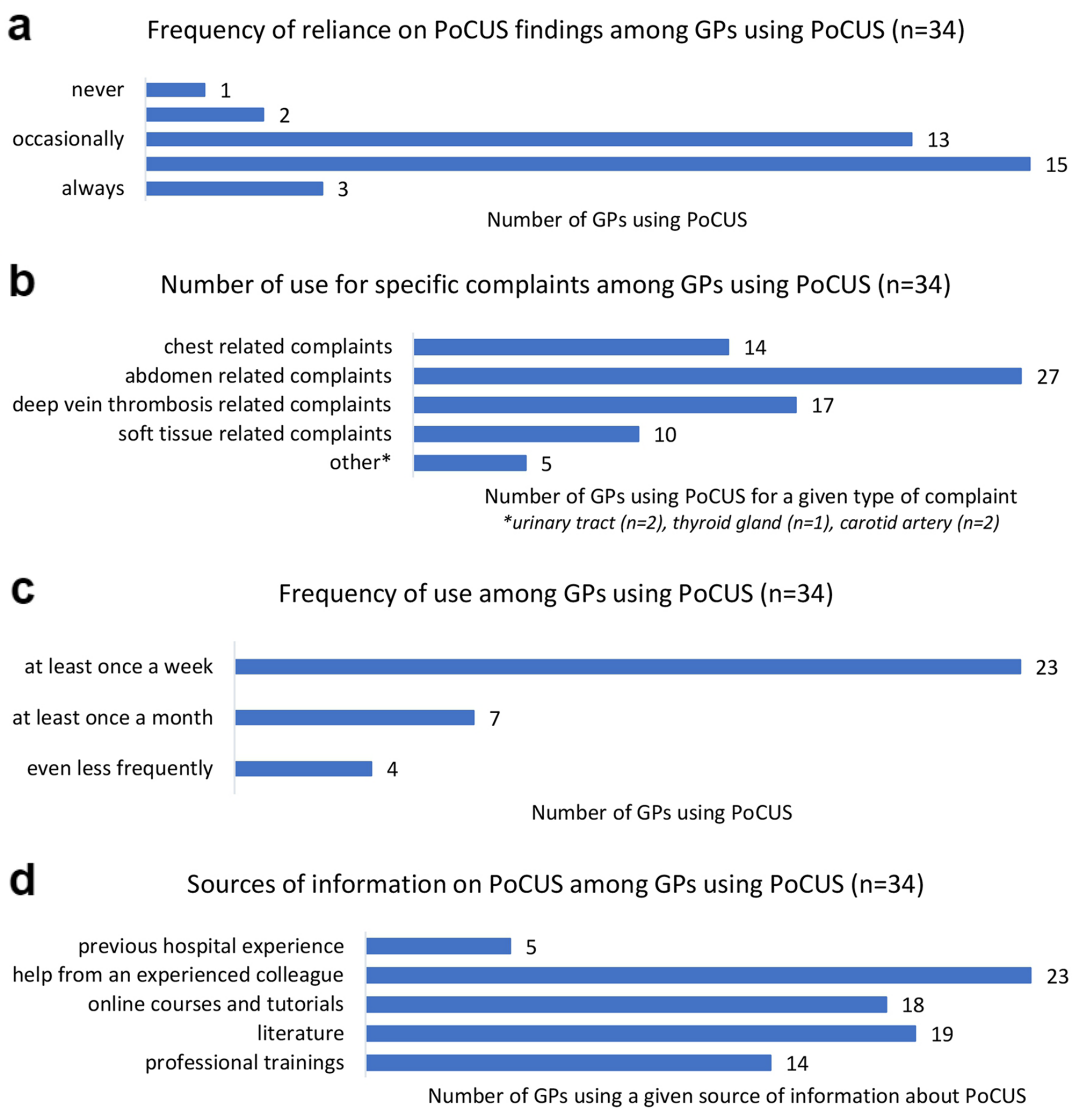
Answers to questions to GPs using PoCUS. **a** Frequency of reliance on PoCUS findings among GPs using PoCUS, **b** Number of use for specific complaints among GPs using PoCUS, **c** Frequency of use among GPs using PoCUS, **d** Sources of information on PoCUS among GPs using PoCUS

The mean age of the GPs was 53.8 ± 11.1 years, with 54.9% (n = 228) of patients being females and 45.1% (n = 187) males. A significant association was seen between the GPs’ age and their willingness to implement PoCUS (*p* = 0.02), as well as to complete PoCUS training (*p* < 0.0001). The mean duration of practice was 19.5 ± 11.9 years, which was also statistically associated with willingness to participate in PoCUS training (*p* = 0.0011). A total of 23.9% (n = 99) of GPs were in mixed (adult and pediatric) care, while 76.1% (n = 316) were in adult care. Overall, 8.2% (n = 34) of the GPs used bedside ultrasonography. For these 34 participants, we asked additional questions about the use of PoCUS, as shown in Fig. 1.

**Fig. 2:**
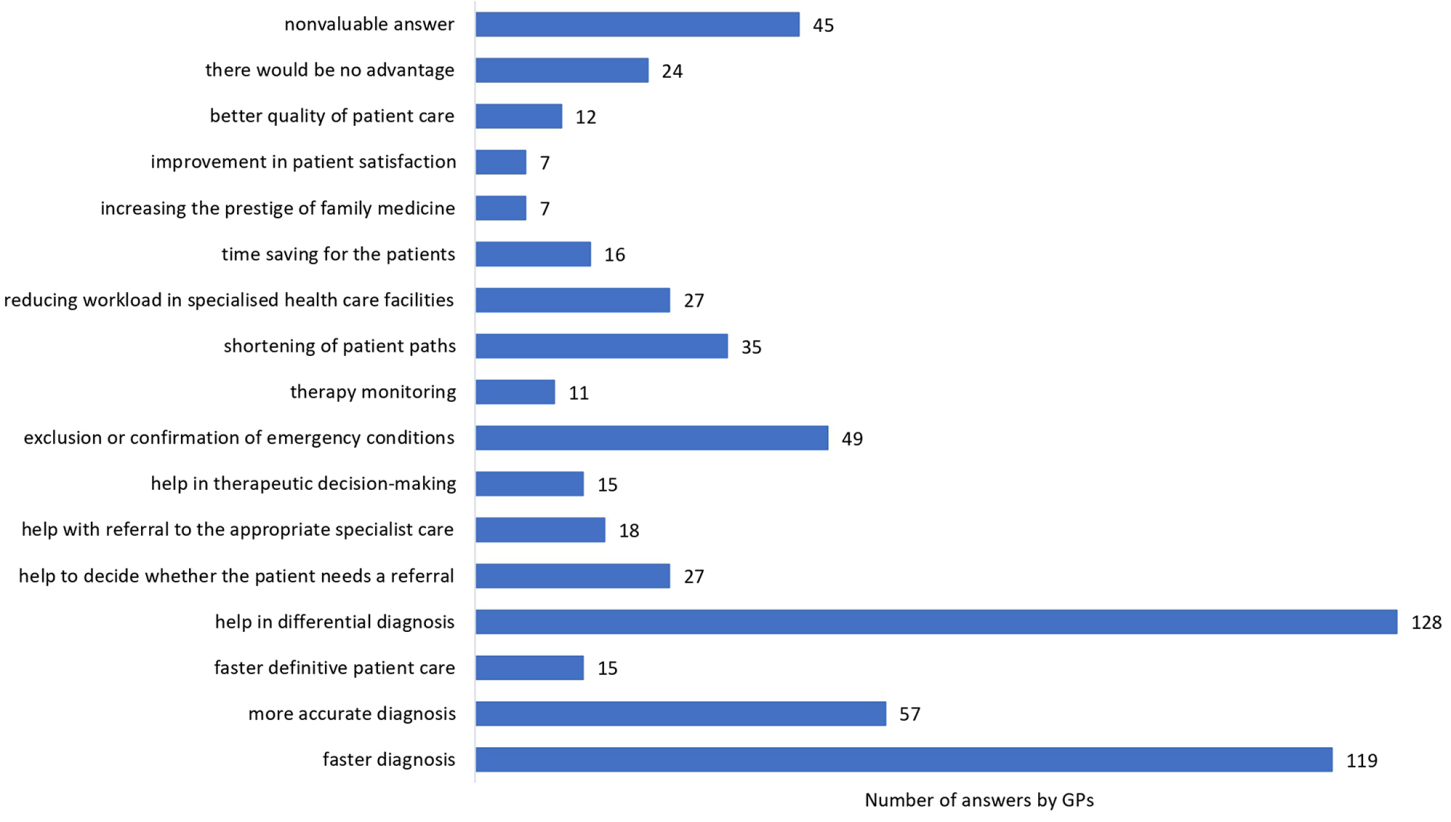
The advantages of point-of-care ultrasonography as expressed by GPs

**Fig. 3:**
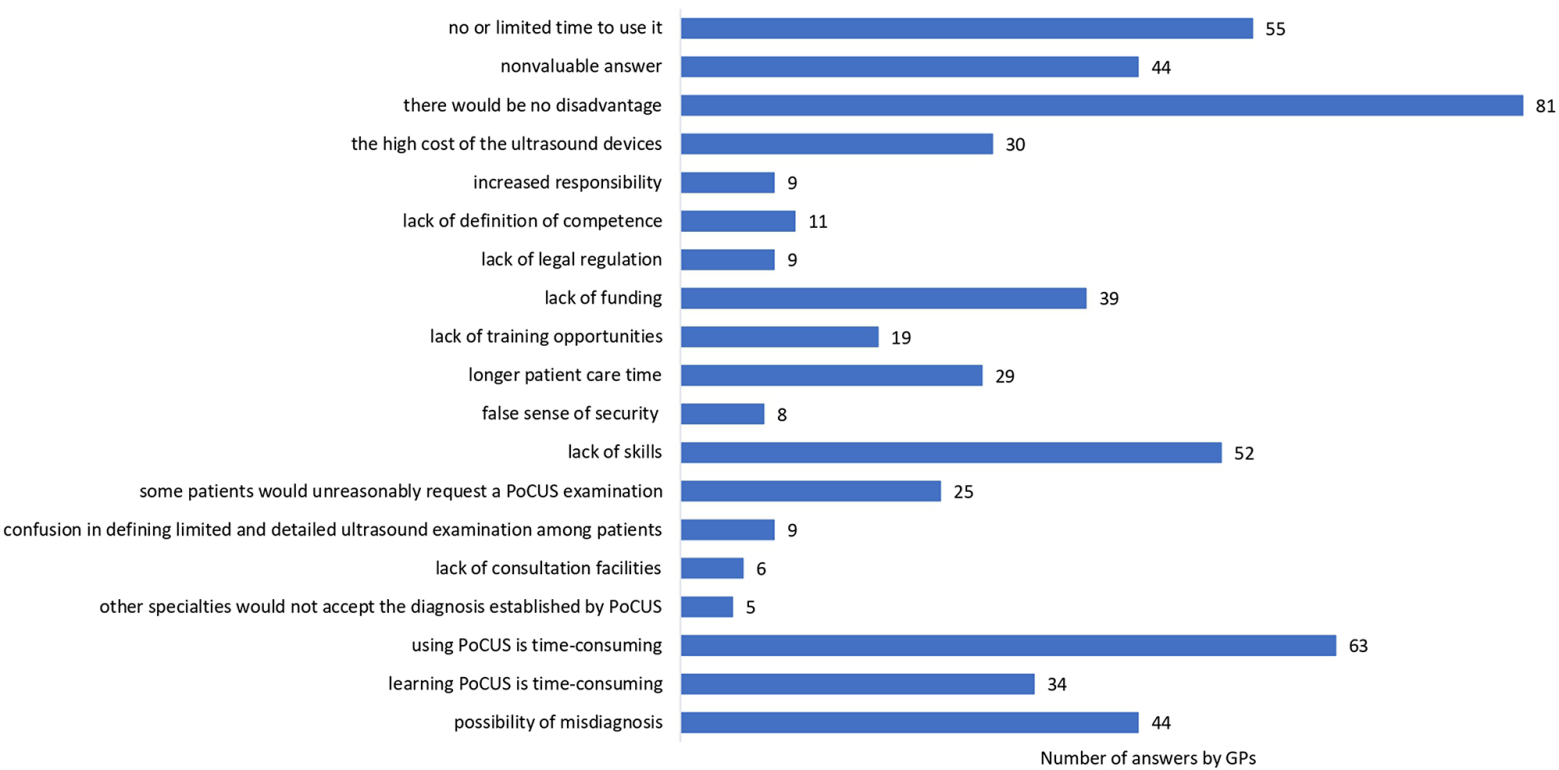
The disadvantages of point-of-care ultrasonography as expressed by GPs

Only 7.2% of GPs (n = 30) rated their knowledge of PoCUS as good, 33.3% limited (n = 138) and 59.5% none (n = 247). Assuming ideal conditions (training, qualification, clear legal and funding aspects, sufficient time to use), 95.4% of GPs (n = 396) believed that PoCUS would contribute to primary care in Hungary, while 4.6% (n = 19) thought the opposite. A total of 82.2% of GPs (n = 342) advocated PoCUS training for family medicine resident doctors. If available, 80.2% (n = 333) would complete PoCUS training, with 59.8% of GPs being confident in finding time for daily use, while 22,9% (n = 95) were uncertain about this question. A total of 88.9% of GPs (n = 369) stressed the need to develop and implement legislation and guidelines. A total of 88.7% (n = 368) of GPs anticipated a positive impact on patient satisfaction. If the ideal circumstances listed above were provided, 79.3% of GPs (n = 329) would incorporate PoCUS into daily practice. The last two questions about the advantages and disadvantages of PoCUS as expressed by GPs are illustrated in Figs. 2 and 3, which were open questions with no response limit. The potential associations between the demographic data of GPs and answers given are presented in Table 1.

**Table 1:** The potential associations between the demographic data and answers given by GPs. A p-value of < 0.05 was considered to be statistically significant

First variable	Second variable	Responses with numbers and frequencies	*p*-value
How familiar are you with Point-of-care ultrasound techniques?	GPs age	„I am familiar with these techniques well”: n = 30 (7.2%) • *< 50 years of age: n = 14 (46.7%)*	0.51
How familiar are you with Point-of-care ultrasound techniques?	Type of GPs practice*	„I am familiar with these techniques well”: n = 30 (7.2%) • *adult care: n = 18 (60.0%)*	0.09
Do you use an ultrasound machine in your daily work?	Type of GPs practice	„Yes”: n = 34 (8.2%) • *adult care: n = 24 (70.6%)*	0.41
Could you make time for using PoCUS during your daily work?	Type of GPs practice	„Yes”: n = 248 (59.8%) • *adult care: n = 73.8%*	0.33
Would you incorporate PoCUS in your daily practice under ideal circumstances (qualification, legal regulation, etc.)?	Type of GPs practice	„Yes”: n = 329 (79.3%) • *adult care: n = 251 (76.3%)*	0.38
Would you incorporate PoCUS in your daily practice under ideal circumstances (qualification, legal regulation, etc.)?	GPs age	„Yes”: n = 329 (79.3%) • *< 50 years of age: n = 132 (40.1%)*	**0.02**
If you had the opportunity, would you take a qualifying training course?	GPs age	„Yes”: n = 333 (80.2%) • *< 50 years of age: n = 136 (40.1%)*	**< 0.0001**
If you had the opportunity, would you take a qualifying training course?	Type of GPs practice	„Yes”: n = 333 (80.2%) • *adult care: n = 136 (76.0%)*	0.98
How important would you consider it to develop and implement detailed legislation and professional guidelines (e.g. protocols, quality assurance standards) for PoCUS?	GPs age	„Important”: n = 369 (88.9%) • *< 50 years of age: n = 136 (36.9%)*	0.87 (with Fisher’s exact test)
How important would you consider it to develop and implement detailed legislation and professional guidelines (e.g. protocols, quality assurance standards) for PoCUS?	Type of GPs practice	„Important”: n = 369 (88.9%) • *adult care: n = 280 (75.9%)*	0.17 (with Fisher’s exact test)
How familiar are you with Point-of-care ultrasound techniques?	Time of practice	„I am familiar with these techniques well”: n = 71 (17.1%) • *< 20 years of practice: n = 35 (49.3%)*	0.22
Do you use an ultrasound machine in your daily work?	Time of practice	„Yes”: n = 34 (8.2%) • *< 20 years of practice: n = 16 (47.1%)*	0.67
Would you incorporate PoCUS in your daily practice under ideal circumstances (qualification, legal regulation, etc.)?	Time of practice	„Yes”: n = 329 (79.3%) • *< 20 years of practice: n = 173 (52.6%)*	0.11
If you had the opportunity, would you take a qualifying training course?	Time of practice	„Yes”: n = 333 (80.2%) • *< 20 years of practice: n = 179 (53.8%)*	**0.0011**
How important would you consider it to develop and implement detailed legislation and professional guidelines (e.g. protocols, quality assurance standards) for PoCUS?	Time of practice	„Important”: n = 369 (88.9%) • *< 20 years of practice: n = 184 (49.9%)*	0.51

*Type of practice means it is either mixed (child and adult) or exclusively adult care

Based on the latest findings of the Hungarian Central Statistical Office, a comparative analysis has been made to assess the differences between the Hungarian general GP population and the participating physicians. The number of GPs in Hungary was 4427 in the year of 2022, however data on the number of active practices is not available. The GP participants (n = 415) had a mean age of 53.8 ± 11.1 years, while according to data from the Hungarian Central Statistical Office, the mean age of GPs is 58 years, based on the latest analysis [19]. No official data on sex distribution is publicly available. The difference in age suggests that our sample might not fully represent the elderly Hungarian GPs, presumably because this generation is less present on the social media platforms where this questionnaire was shared. While the study provides valuable insights into the perspectives of younger GPs, the above details limit the generalisability of the results.

### Results of the questions answered by patients

The patients (n = 693) had a mean age of 45.5 ± 12.3 years, with 95.24% of them being females and 4.76% males. Regarding residence, 30.2% lived in Budapest, 25.3% in a large city (not including the capital), 25.4% in a small town, and 19.1% in smaller communities. A total of 55.3% of patients had a higher level of education, 44.0% had a secondary education. Patients with a higher level of education would be more willing to pay for a PoCUS examination (*p* = 0.024). Among those patients (n = 427), would pay an average of 5.000 HUF (± 4.623 HUF) for the service. 45.9% of patients would allow their GP to perform PoCUS without qualifications, but the proportion of patients accepting the examination increased to 99.4% if the GP has a formal training in ultrasonography. In the case of an untrained GP, 48.5% (n = 336) of patients would not completely believe the diagnosis. In contrast, 93.4% of patients (n = 647) would believe the result established by a certified GP. In this study, a total 93.8% of patients (n = 650) considered it essential to be trained in bedside ultrasound, and 94.6% found it important to receive detailed information about the examination. The potential associations between the demographic data and answers given are presented in Table 2.

**Table 2:** The potential links between the demographic data and answers given by patients. A p-value of < 0.05 was considered to be statistically significant

First variable	Second variable	Responses with numbers and frequencies	*p*-value
How important is it for you that your GP is qualified for PoCUS?	Level of education	„I consider it important”: n = 578 (83.4%) • *higher level of education: n = 323 (55.9%)* • *secondary education: n = 250 (43.3%)*	0.43
How important is it for you that your GP is qualified for PoCUS?	Type of residence	„I consider it important”: n = 578 (83.4%) • *capital*,* large city or county seat: n = 328 (56.7%)* • *small town: n = 138 (23.9%)* • *municipality or village: n = 112 (19.4%)*	0.22
Would you allow your GP to perform PoCUS on you without a qualification?	Level of education	„Yes”: n = 318 (45.9%) • *higher level of education: n = 172 (54.1%)* • *secondary education: n = 144 (45.3%)*	0.77
Would you allow your GP to perform PoCUS on you without a qualification?	Type of residence	„Yes”: n = 318 (45.9%) • *capital*,* large city or county seat: n = 169 (53.1%)* • *small town: n = 86 (27.1%)* • *municipality or village: n = 63 (19.8%)*	0.16
Would you believe your GP’s PoCUS report if he or she is not qualified?	Level of education	„Yes”: n = 144 (20.8%) • *higher level of education: n = 77 (53.5%)* • *secondary education: n = 65 (45.1%)*	0.72
Would you believe your GP’s PoCUS report if he or she is not qualified?	Type of residence	„Yes”: n = 144 (20.8%) • *capital*,* large city or county seat: n = 72 (50.0%)* • *small town: n = 40 (27.8%)* • *municipality or village: n = 32 (22.2%)*	0.35
Would you pay for a PoCUS examination performed by your GP?	Level of education	„Yes”: n = 427 (61.6%) • *higher level of education: n = 255 (59.7%)* • *secondary education: n = 168 (39.3%)*	**0.024**
Would you pay for a PoCUS examination performed by your GP?	Type of residence	„Yes”: n = 427 (61.6%) • *capital*,* large city or county seat: n = 232 (54.3%)* • *small town: n = 108 (25.3%)* • *municipality or village: n = 87 (20.4%)*	0.28
If you would pay, then how much? (n = 427)	Level of education	Less than 5000 HUF: n = 71 (16.6%) • *higher level of education: n = 38 (53.5%)* • *secondary education: n = 31 (43.7%)* Between 5000 and 10,000 HUF: n = 296 (69.3%) • *higher level of education: n = 177 (59.8%)* • *secondary education: n = 117 (39.5%)*	0.26
If you would pay, then how much? (n = 427)	Type of residence	Less than 5000 HUF: n = 71 (16.6%) • *capital*,* large city or county seat: n = 41 (57.7%)* • *small town: n = 20 (28.2%)* • *municipality or village: n = 10 (14.1%)* Between 5000 and 10,000 HUF: n = 296 (69.3%) • *capital*,* large city or county seat: n = 158 (53.4%)* • *small town: n = 75 (25.3%)* • *municipality or village: n = 63 (21.3%)*	0.58

**Fig. 4:**
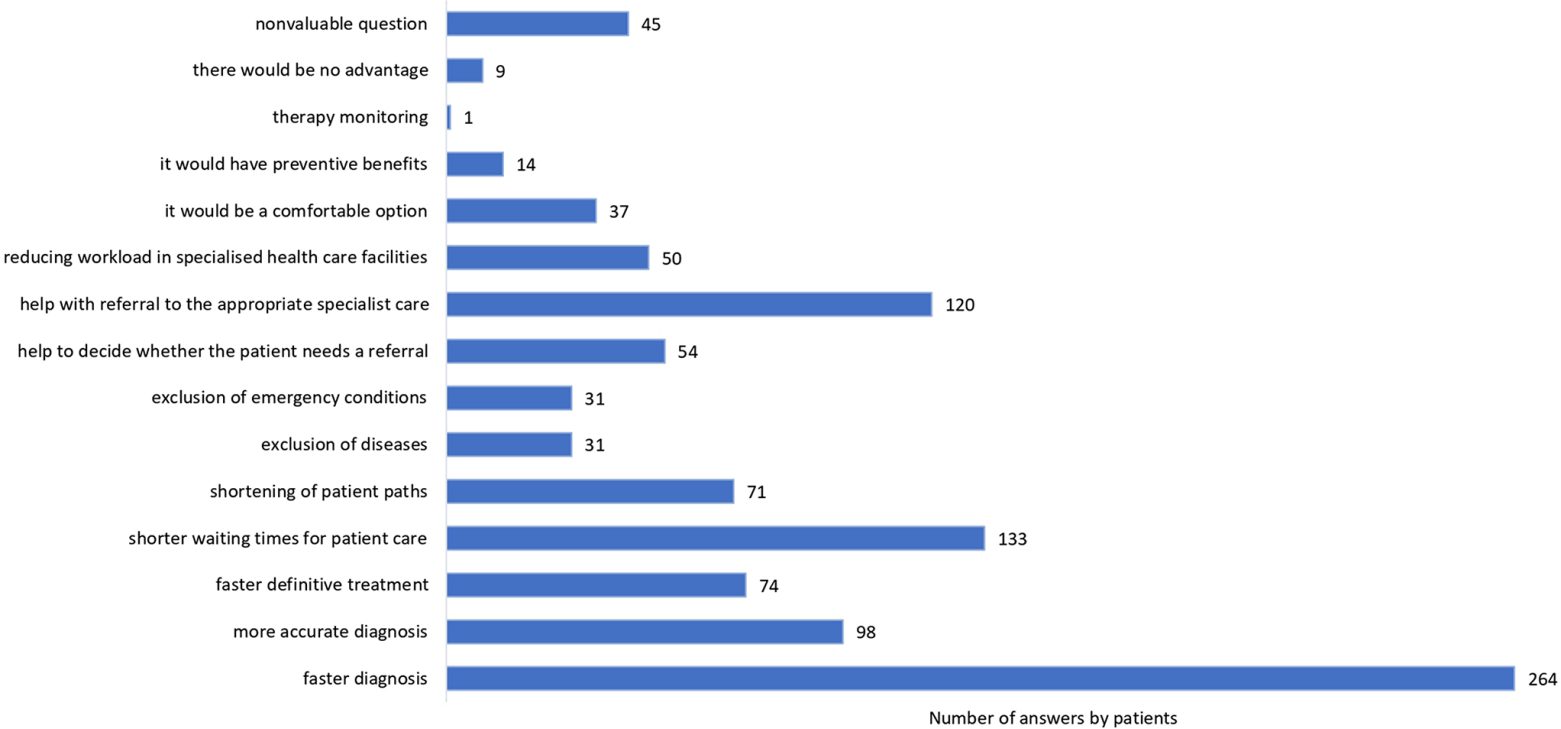
The advantages of point-of-care ultrasonography as expressed by patients

**Fig. 5:**
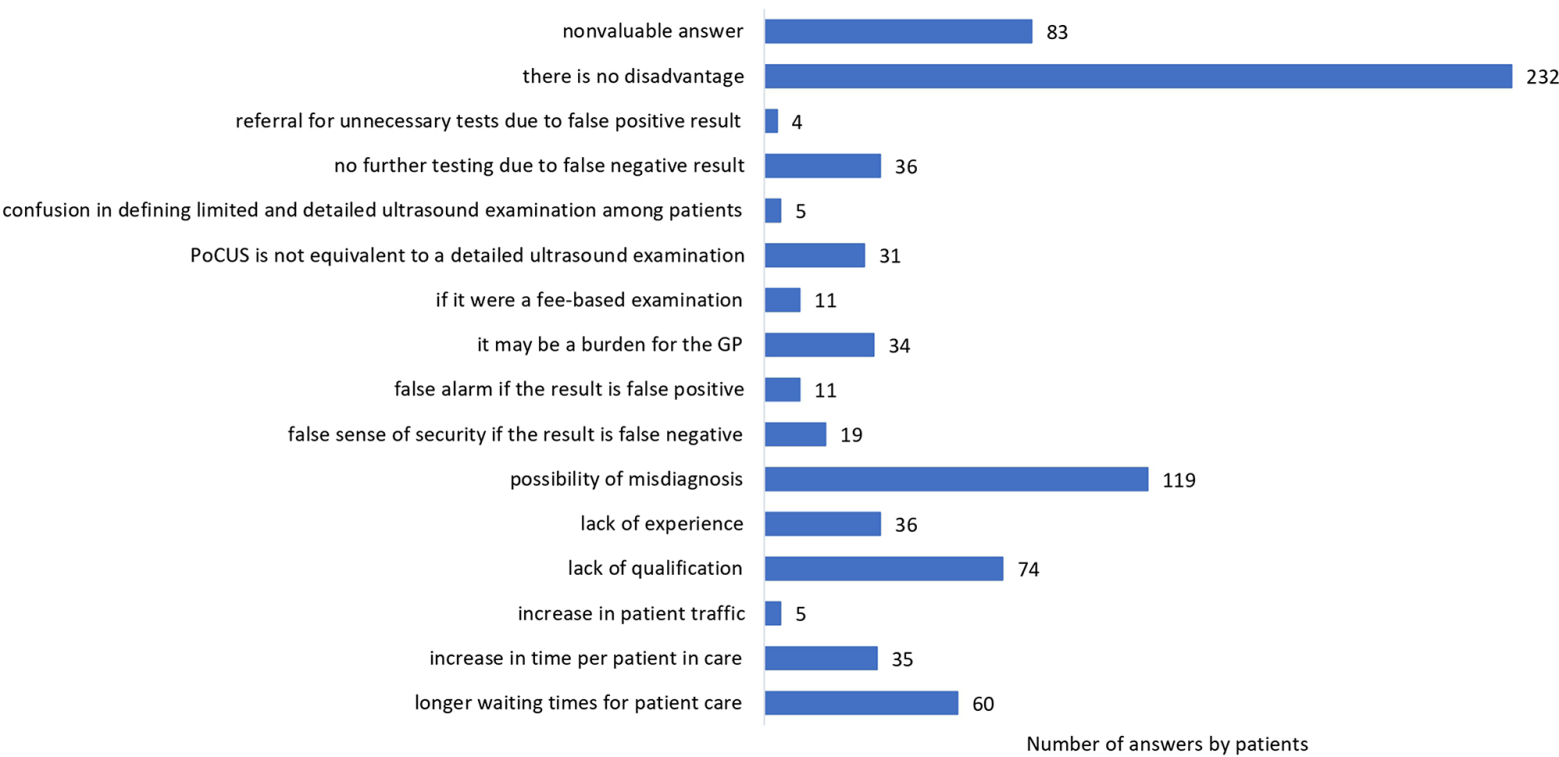
The disadvantages of point-of-care ultrasonography as expressed by patients

In terms of patient satisfaction, 99.1% of patients would be satisfied if their GP were certified in PoCUS. The advantages and disadvantages of PoCUS, as expressed by patients, are illustrated in Figs. 4 and 5, which were open questions with no response limit.

## Discussion

### Main findings

Patients and GPs living and working in Hungary were surveyed on their attitudes and experiences toward PoCUS in primary care. Although the number of GPs using PoCUS on a daily basis is currently very low, practitioners and patients demonstrated a great interest and willingness to introduce bedside ultrasound. For GPs, the willingness to implement and learn PoCUS is significantly influenced by age, which suggests that younger GPs may be more open to adapting to modern technology. The responses showed higher trust and acceptance from patients, especially if their GPs are qualified to perform PoCUS. A statistically significant relationship was found between patients’ willingness to pay for the examination and their level of education, suggesting that patients who may have a better financial background due to their higher level of education are more willing to pay the availability of high-quality healthcare.

### Comparison with the literature

The process of adapting PoCUS to primary care is occurring at very different paces worldwide. In the USA, despite an increasing number of states including PoCUS in the training of family medicine residents, the rate of use by specialists remains low. The solution may lie in addressing the immaturity of the training infrastructure through targeted investments [20]. Furthermore, cooperation between family medicine departments to share best practices and continuity in procuring equipment and establishing a financing scheme are essential for further expansion [11]. Our study has shown that these are the main areas that Hungary needs to improve. A previous study revealed that family medicine residents and GPs showed high interest and importance to PoCUS [21]. According to the results of our research, these findings are in line with the views of Hungarian GPs. PoCUS began to gain ground in the primary care of more European countries but still not in sufficient quantities. In Denmark, an increasing number of GPs are using PoCUS despite the lack of evidence-based guidelines for its appropriate use. For this reason, Danish GPs who require PoCUS have developed guidelines for themselves, as reported in an article in 2019 [22]. In the same year, Danish, Norwegian, Swedish, and Finnish GPs experienced in PoCUS developed a list of ultrasound examinations that could be used as a guideline in primary care, which could form the basis for future training [23]. In Catalonia, Spain, based on the consensus of GPs experienced in PoCUS, a training curriculum was developed for the main indications of PoCUS in primary care, in which competence levels were also defined [24]. The Danish and Spanish consensus-building methods could be good examples for the development of a detailed curriculum of PoCUS in Hungarian primary care, given our findings that there is a strong need to develop it. However, knowing the risks of using PoCUS without following existing professional guidelines is vital. In Norway, there was a fourfold increase in the number of GPs using PoCUS between 2009 and 2016, with 30% of them using the device by 2016 [25]. Similar to this study, in the future, we consider it useful to survey the number of PoCUS users among GPs in Hungary, where our current survey results would be the basis for comparison. In Slovenia, a small number of GPs included in a questionnaire used PoCUS for a wide range of indications, while at the same time, there was a significant number of disadvantages. The disadvantages mentioned by GPs were related to the organisation of examinations, the immature nature of training and the lack of funding [26]. The same problems currently exist in Hungary and need to be solved. A systematic review demonstrated that GPs are able to use PoCUS safely in a wide range of clinical settings, given a certain level of pretest probability [27]. One study revealed that using PoCUS in 3 out of 4 consultations influenced the diagnostic process and clinical decision-making of GPs [13]. The results of these studies and our current survey support the need to address the problems hindering the implementation of PoCUS in Hungary. Our study shows that there is considerable interest among Hungarian GPs and patients in implementing PoCUS, and despite the low rate of its current use, there is a strong willingness to learn and incorporate PoCUS into day-to-day medical practice. The benefits of introducing PoCUS into primary care are also highlighted in the study by Wordsworth et al., including the potential cost-effectiveness of PoCUS, if training and resources are available [28]. Addressing barriers such as insufficient training, time constraints, and limited access to devices is crucial for the successful implementation of PoCUS [20, 21]. Therefore, developing comprehensive and standardised PoCUS training programs and ensuring adequate resource allocation are essential for integrating PoCUS into Hungarian primary care. From the patient’s perspective, a previous study has shown that a significant proportion of the patients involved had an overall positive experience of PoCUS in their GP practice [29]. This result aligns well with the direction of patients’ attitudes in our research, but further in-depth studies are needed to explore patients’ views in more detail.

### Implications for research, clinical practice, education and policy

Our study underlines the demand for targeted interventions in Hungary to address the limited use of PoCUS among GPs. Key implications include developing structured training programs, integrating residency training, and establishing standardised guidelines and regulations. Bridging the gap between willingness and utilisation requires educational efforts addressing technical and perception-related aspects. Lawmakers should focus on legislation and guidelines to support the implementation of PoCUS in Hungarian primary care settings.

### Strengths and limitations

Our research’s strength is its comprehensive approach, gathering the views of providers and beneficiaries to understand PoCUS perspectives in Hungarian primary care. Anonymous online questionnaires on social media enable honest responses, with open-ended questions allowing unrestricted opinions from participants. However, our study has limitations as well, including the involvement of patients and GPs, that has been through closed social media platforms. Older patients and physicians are less represented on these online platforms, which may result in potential biases. The interpretation of our results is further limited by the fact that the sex of the patient population is almost entirely female. However, this observation may indicate that female patients are more involved in health-related issues. The cross-sectional design provides only a snapshot, limiting causal relationships and temporal monitoring.

## Conclusions

Our findings indicate a a significant interest in adapting PoCUS in primary care from both GPs and patients. Based on the fact that a significant proportion of Hungarian GPs are unaware of PoCUS and its indications, it is particularly important to develop educational frameworks, and practical guidelines for the effective incorporation of PoCUS in Hungary.

## Electronic supplementary material

Below is the link to the electronic supplementary material.

Supplementary Material 1

## Data Availability

The datasets used and/or analysed during the current study are available from the corresponding author on reasonable request.

## Abbreviations

EUR: Euro
GP: General Practitioner
HUF: Hungarian Forint
PoCUS: Point-of-Care Ultrasound
SD: Standard Deviation
USA: United States of America
