# Supplementary material for: Is it worth trying? A cross-sectional study on the implementation of point-of-care ultrasound in Hungarian primary care
Source: BMC Prim Care. 2024 Sep 5;25:328. doi: 10.1186/s12875-024-02578-z (PMC11375868; doi:10.1186/s12875-024-02578-z)
Supplement: Supplementary file 1 — Supplementary Material 1 [file 12875_2024_2578_MOESM1_ESM.docx]

**Questionnaires**

The content of the questionnaires was written in Hungarian. Questionnaires were designed in the interface of the *EvaSys* system. This survey-generating interface is used at the University of Szeged for research purposes.

The questionnaires filled in by the participants are available on the university's secure network, therefore they cannot be accessed by unauthorised persons.

The English translation of the questionnaires can be found below:

**Questions of the questionnaire for General practitioners:**

1. **Demographic questions**
2. How old are you? (open question)
3. What is your sex? (single choice)

- Female.
- Male.

1. **Questions about the GP's practice**
2. How many years have you been practising as a general practitioner? (open question)
3. What is the type of your practice? (single choice)

- Mixed practice (medical care for both adults and children).
- Adult practice (medical care for adults only).

1. Do you use an ultrasound machine in your daily work? (single choice)

- Yes.
- No.

*Participants who answered „Yes.” to the previous question were asked the following four questions:*

1. How often do you use ultrasound machine at work? (single choice)

- At least once a week.
- At least once a month.
- Even less frequently.

1. For which symptoms, complaints, physical signs do you usually complement your examinations with an ultrasound scan? (multiple choice)

- Chest.
- Abdomen.
- Veins of lower extremity.
- Soft tissue.
- Other.

1. From what sources do you learn about bedside ultrasound? (multiple choice)

- Previous hospital experience.
- Help from experienced colleague.
- Online courses and tutorials.
- Literature.
- Professional trainings.

1. How often do you rely on the results of the ultrasound scan to make treatment decisions? (single choice)

- Never.
- Rarely.
- Occasionally.
- Frequently.
- Always.

1. How familiar are you with Point-of-care ultrasound techniques (single choice)?

- I am familiar with these techniques well.
- I have limited knowledge.
- I am not familiar with these techniques.

1. Please describe what advantages you currently see in learning and using bedside ultrasound examination techniques in your practice! (open question)
2. Please describe what disadvantages you currently see in learning and using bedside ultrasound examination techniques in your practice! (open question)
3. Under ideal conditions (available training, qualification, clarification of legal and financial aspects, sufficient time), to what extent do you think bedside ultrasound would contribute to the developement of primary care in Hungary? (single choice)

- To a very large extent.
- To a large extent.
- To a medium extent.
- To a small extent.
- Not at all.

1. Do you think it would be necessary to introduce theoretical and practical training in bedside ultrasound in the curriculum of GP residents in Hungary? (single choice)

- Yes.
- No.
- I don’t know.

1. If you had the opportunity, would you take a qualifying training course? (single choice)

- Yes.
- No.
- I don’t know.

1. Could you make time for using PoCUS during your daily work? (single choice)

- Yes.
- No.
- I don’t know.

1. How important would you consider it to develop and implement detailed legislation and professional guidelines (e.g. protocols, quality assurance standards) for PoCUS? (single choice)

- Important.
- Less or not important.

1. What impact do you think the use of bedside ultrasound would have on patient satisfaction with their care? (single choice)

- My patients' satisfaction would improve significantly.
- My patients' satisfaction would improve somewhat.
- My patients’ satisfaction would not change.
- My patients’ satisfaction would be reduced.
- My patients’ satisfaction would be significantly reduced.

1. Would you incorporate PoCUS in your daily practice under ideal circumstances (qualification, legal regulation, etc.)? (single choice)

- Yes.
- No.
- I don’t know.

**Questions of the questionnaire for Patients:**

1. **Demographic questions**
2. How old are you? (open question)
3. What is your sex? (single choice)

- Female.
- Male.

1. What is your educational level? (single choice)

- Completed or less than 8 years of primary school.
- Completed high school or vocational secondary school.
- College or university degree.

1. What is the type of your residence? (single choice)

- Capital.
- Large city or county seat.
- Small town.
- Municipality or village.

1. **Questions related to the implementation and use of PoCUS in GP practices**
2. Would you allow your GP to perform PoCUS on you without a qualification? (single choice)

- Yes.
- No.
- I don’t know.

1. Would you allow your GP to perform PoCUS on you with a qualification? (single choice)

- Yes.
- No.
- I don’t know.

1. How important is it for you to have an ultrasound machine in your GP’s practice? (single choice)

- I consider it important.
- I consider it less or not important.

1. How important is it for you that your GP is qualified for PoCUS? (single choice)

- I consider it important.
- I consider it less or not important.

1. If your GP wanted to perform a bedside ultrasound scan on you in the practice in addition to the usual tests, and your GP were qualified to do so, how satisfied would you be with the care? (single choice)

- I would be very satisfied.
- I would be satisfied.
- I would be less satisfied.
- I would not be satisfied.

1. If your GP wanted to perform a bedside ultrasound scan on you in the practice in addition to the usual tests, and your GP were not qualified to do so, how satisfied would you be with the care? (single choice)

- I would be very satisfied.
- I would be satisfied.
- I would be less satisfied.
- I would not be satisfied.

1. Do you consider it necessary to train GPs in ultrasound scanning in order to improve primary care? (single choice)

- Yes.
- No.
- I don’t know.

1. Would you be willing to pay for an ultrasound scan performed by your qualified GP if this medical activity was included in the fee schedule for reimbursable GP services? (single choice)

- Yes.
- No.
- I don’t know.

*Participants who answered „Yes.” to the previous question were asked the following question:*

1. If you would pay, then how much? (open question)
2. What do you think would be the advantages of having your GP use an informative, limited value ultrasound scan on you? Please write your opinion! (open question)
3. What do you think would be the disadvantages of having your GP use an informative, limited value ultrasound scan on you? Please write your opinion! (open question)
4. Do you think it is important for your GP to give you detailed information about the ultrasound scan he or she performs? (single choice)

- I consider it important.
- I consider it less or not important.

1. Would you believe your GP’s PoCUS report if he or she is qualified? (single choice)

- Yes.
- No.
- I don’t know.

1. Would you believe your GP’s PoCUS report if he or she is not qualified? (single choice)

- Yes.
- No.
- I don’t know.
